# Effects of red light on sleep and mood in healthy subjects and individuals with insomnia disorder

**DOI:** 10.3389/fpsyt.2023.1200350

**Published:** 2023-08-24

**Authors:** Rong Pan, Guimei Zhang, Fangyi Deng, Weifeng Lin, Jiyang Pan

**Affiliations:** ^1^Department of Psychology, The Third People’s Hospital of Zhaoqing, Zhaoqing, Guangdong Province, China; ^2^Department of Psychiatry, Sleep Medicine Center, First Affiliated Hospital of Jinan University, Guangzhou, Guangdong Province, China; ^3^Department of Neurology, Dongguan People’s Hospital (Affiliated Dongguan People’s Hospital, Southern Medical University), Guangzhou, Guangdong Province, China

**Keywords:** insomnia disorder, red light, positive and negative emotion, alertness, polysomnography

## Abstract

**Introduction:**

This study aimed to determine the influence of red light on objective sleep and the relationship between mood and sleep among individuals with insomnia disorder (ID).

**Method:**

57 individuals with insomnia symptoms and 57 healthy participants were randomly divided into three groups (red- and white-light groups, and the black control group), which received different light treatments for 1 h before bedtime. The emotions and subjective alertness of participants were evaluated using Positive and Negative Affect Schedule scales (PANAS) and Karolinska Sleepiness Scale (KSS), their sleeping data were recorded using polysomnography (PSG).

**Result:**

The negative emotion scores were higher in the healthy subject-red light (HS-RL) group than in the HS-white light (WL) and HS-black control (BC) groups (*p* < 0.001). The anxiety and negative emotion scores were higher in the ID-RL group than in the ID-WL and ID-BC groups (*p* = 0.007 and *p* < 0.001, respectively). The KSS scores were lower in the RL group than in the WL and BC groups for both HS and ID group (both *p* < 0.001). The SOL was shorter in the HS-RL group than in HS-WL group (*p* = 0.019). Compared with the HS-BC group, the HS-RL group had an increase in microarousal index (MAI) and N1% (*p* = 0.034 and *p* = 0.021, respectively), while the total sleep time (TST) and sleep efficiency (SE) decreased (*p* = 0.001 and *p* < 0.001, respectively). Compared with the ID-WL group, the SOL was shorter in the ID-RL group (*p* = 0.043), while TST, SE, number of microarousals (NMA), and numbers of cycles of REM period were increased (*p* = 0.016, *p* = 0.046, *p* = 0.001, and *p* = 0.041, respectively). Compared with the ID-BC group, the ID-RL group had increases in the SOL, WASO, and the numbers of cycles and NMA in REM period (*p* = 0.038, *p* = 0.005, *p* = 0.045, and *p* = 0.033, respectively), and a decrease in SE (*p* = 0.014). The effects of ID-WL (vs. ID-RL group) and ID-BC (vs. ID-RL group) on SOL were mediated by negative emotions (mediating effects were − 37.626 and − 33.768, respectively).

**Conclusion:**

Red light can increase subjective alertness, anxiety, and negative emotions in both healthy subjects and people with ID, which can affect sleep directly or indirectly via the mediating effect of negative emotions.

## 1. Introduction

It is well known that light can affect the circadian rhythms of animals, which in turn regulates their sleep-awake cycle (1). In the natural world, circadian rhythms are often mediated by the change between day and night created by the rotation of the Earth. However, for the past 150 years the natural photoperiod has been disrupted by the presence of artificial light, and global light pollution has grown rapidly (2, 3). Light pollution can be divided into three categories: white- and colored-light pollution, and sky glow. Studies have found that exposure to high-intensity sky glow at night can delay sleep onset to varying degrees, increase the number of nighttime awakenings, reduce total sleep time, and change the proportions of Rapid Eye Movements (REM) and Non-Rapid Eye Movements (NREM) (4). The increase in light pollution interferes with normal sleep, and insomnia is becoming more common, with data indicating that insomnia disorder (ID) prevalence among adults has reached 9.2% in China (3, 5). Controlling lighting at night is therefore of great importance to people.

Red light caught our attention because light regulates the sleep-awake cycle via the intrinsically photosensitive retinal ganglion cells (ipRGCs), and the melanopsin cells expressed in ipRGCs are the least sensitive to red light, which might disrupt the original sleep-awake rhythm of the organism less than white light does (6, 7). Red light is often used as a light source for nighttime illumination in laboratories, and some scholars believe that artificial red light can reduce sleep-awake cycle disruption at night and improve sleep more than ordinary white light (8–10). Some studies have also suggested that ordinary light at night can be replaced with red light to help sleep (9, 11). However, there are also some uncertainties regarding red light, such as it being more likely to induce arousal and negative emotional experiences (12), and to increase arousal levels and alertness (12–14). Increased excitability, high arousal levels, and anxiety are all detrimental factors to sleep onset. Red light was also found to significantly increase daytime alertness in subjects and both subjective and objective alertness in those working night shifts (15, 16), but some studies suggested that red light may inhibit objective alertness (13, 14). Red light may also influence melatonin secretion, which affects sleep (17, 18). Some researchers have also suggested that light pollution at night can indirectly affect human mood by affecting sleep quality, but the complex mechanisms remain unclear (2, 19), and so further exploring the relationships among red light, sleep, and mood is reasonable.

In summary, studies on how light affects circadian rhythms and sleep have expanded to explore the role of monochromatic light, but we are aware of few objective studies with a single-blind randomized design have assessed the impact of red light on sleep structure. We therefore aimed to test the hypothesis that red light affects sleep structure by influencing alertness, mood directly and indirectly, and reveal how red-light impacts sleep by exploring the interrelationship between red light, sleep, and emotion, with the aim of providing a reference for the prevention and alleviation of ID and its complications.

## 2. Methods

### 2.1. Participants

This study recruited and enrolled 123 participants aged 18–65 years from Guangzhou city. None of the participants were patients of the researchers. The primary inclusion criterion for participants with insomnia symptoms were self-reported symptoms of insomnia for >3 months based on the diagnostic criteria in the fifth edition of the Diagnostic and Statistical Manual of Mental Disorders (DSM-5). Insomnia was identified in the study participants using validated symptom questionnaires: Insomnia Severity Index (ISI) (20) score ≥ 14, and Pittsburgh Sleep Quality Index (PSQI) (21) score ≥ 8. Healthy participants enrolled in the study did not have insomnia or emotional symptoms. The following exclusion criteria were applied: comorbid cerebral organic diseases and other serious physical diseases, or chronic eye diseases and using photosensitization drugs; comorbid mental disorders or family history of mental disorders (e.g., bipolar disorders, dependence on, or abuse of psychoactive substances); coexistence of other sleep disorders such as sleep-related breathing and movement disorders; using any hypnotics, antidepressants, antipsychotics, or antihistamines during the previous 2 weeks; traveling across time zones or working in shifts during the past week (replacement of night and day shifts); drinking excessive amounts of tea, wine, or coffee; or pregnant and lactating.

After screening, two patients with obstructive sleep apnea syndrome, three with periodic limb movement disorder, and four who dropped out of the study (three patients could not participate in the intervention, and one refused to receive the lighting intervention) were excluded. The procedure was reviewed and approved by the ethics committees of the First Affiliated Hospital of Jinan University and followed the design of the CONSORT extension for randomized trials of nonpharmacological treatment (22). Written informed consent was obtained from all enrolled participants.

### 2.2. Procedure

**Figure 1:**
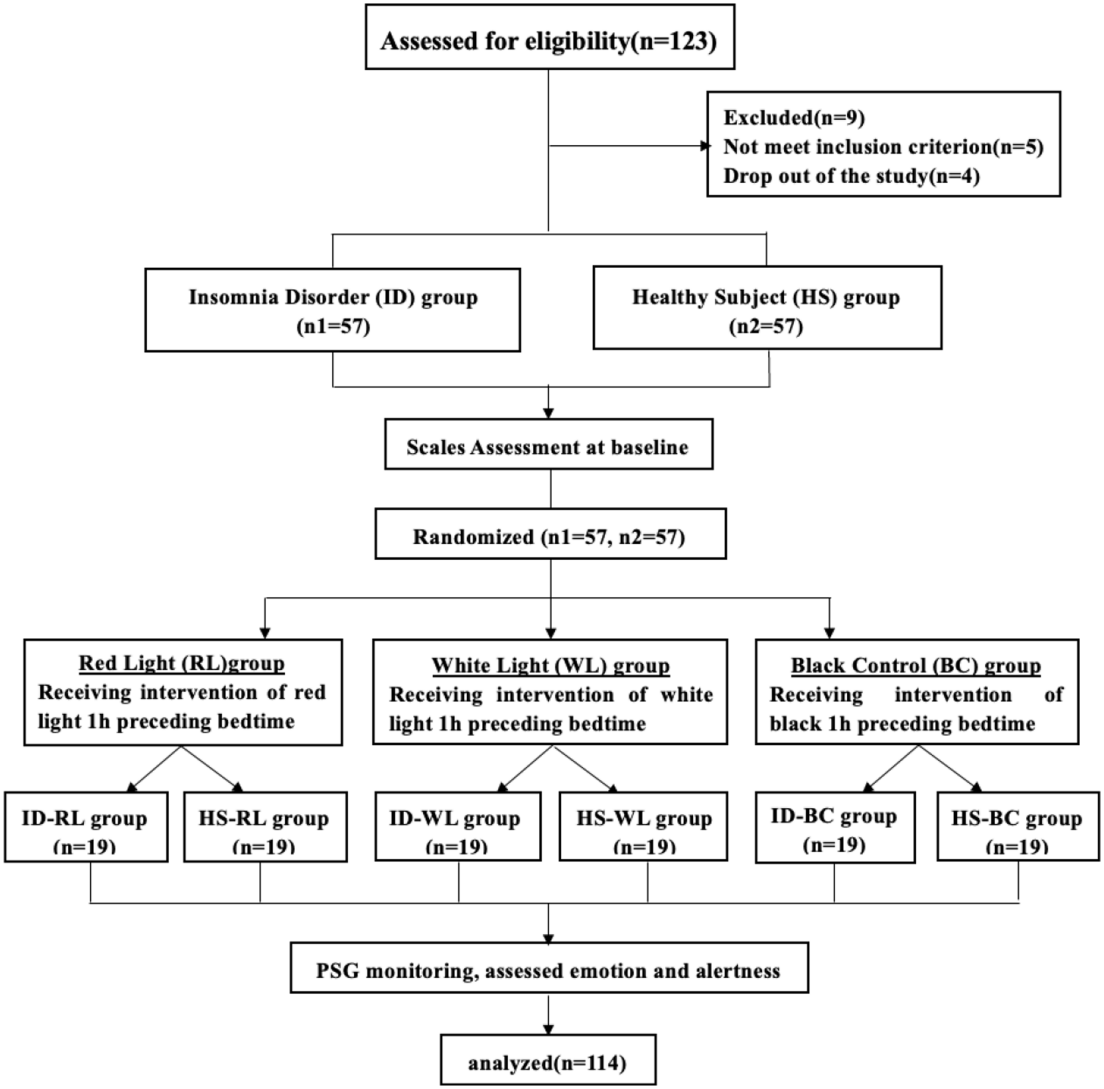
CONSORT enrollment flow diagram. CONSORT, consolidated standards of randomized trials.

The study had a single-blind randomized design. The intervention consisted of receiving three adjustable LED lighting panels (60 cm long and 60 cm wide). Spectral ranges were a white illuminance of 10 ~ 1,400 lx, a wavelength range of 400 ~ 725 nm, and a peak output at 480 nm, and a red illuminance of 10 ~ 250 lx, a wavelength range of 620 ~ 760 nm, and a peak output at 625 nm. According to the standard value of general activity lighting in the bedroom in the Architectural Lighting Design Standards (no. GB 50034–2013) (23), the light intensity in the activity area of a person should be maintained at about 75 lx in a 0.75-m horizontal plane. At baseline, participants completed Mini-International Neuropsychiatric Interview (M.I.N.I.), general condition assessments, and subjective questionnaires to evaluate sleep quality and insomnia severity (ISI and PSQI), and their emotion status [Self-Rating Depression Scale (SDS) (24), and Self-Rating Anxiety Scale (SAS) (25)]. For the intervention, the random envelope method was then used for random grouping. Each lighting group scheme was placed in an opaque envelope with a code written on the outside. After the initial screening of the study subjects on the first night, unique identification numbers were assigned to the participants. Subsequently, the intervention was conducted according to the lighting method specified inside the envelope. Participants were randomly assigned to the red light (RL) group, white light (WL) group, and black control (BC) group, and received light intervention for 1 h before bedtime. Their mood [SAS, SDS, and Positive and Negative Affect Schedule (PANAS) (26)], alertness [Karolinska Sleepiness Scale (KSS) (27)], and objective sleeping structure [polysomnography (PSG)] were assessed (Figure 1).

### 2.3. Outcome measures

Polysomnography parameters were the primary outcome. All study participants underwent two nights (one screening night and one intervention night) of PSG monitoring in the sleep laboratory. PSG data were collected using Compumedics (Australia), and electrodes were placed according to the 10–20 international electrode placement system. We used electrogram electrodes (F4-M1, F3-M2, C4-M1, C3-M2, O2-M1, and O1-M2), eye movement electrodes (LOC-M1 and ROC-M2), mental muscle electrodes (Chin1 and Chin2), ECG electrodes (ECG1 and ECG2), two anterior tibialis EMG (right and left legs), and pulse oximetry, respiration-related electrodes (nasal–oral thermistor, nasal pressure sensors, and thoracic and abdominal impedance). Electrode attachments for all PSG was performed in the sleep laboratory by trained technicians, and sleep stage scoring was assessed by two experienced PSG technologists who had previously completed concordance programs (agreement for epoch-by-epoch comparisons of sleep stage scoring and event scoring) and were blinded to group allocation. The main indexes were sleep onset latency (SOL), total sleep time (TST), sleep efficiency (SE), number of awakenings (NWAK), wakefulness after sleep onset (WASO), N1, N2, N3, and REM period percentages (N1， N2， N3，and R%), number of microarousals (NMA) and microarousal index (MAI), and indicators relating to the REM period (number of cycles, latency, and microarousal times and index).

The secondary outcomes included PANAS and KSS scores. The PANAS is a 20-item, five-point scale, with 10 factors used to represent positive emotions (interested, focused, enthusiastic, mentally active, alert, energetic, proud, determined, inspired, and energetic) and negative emotions (upset, restless, restless, guilty, hostile, nervous, irritable, shy, fearful, and scared). A higher PANAS score indicates stronger emotional expression. The KSS is a nine-item, nine-point scale that requires subjects to review their psychophysiological state over the past 10 min and select the item that reflects their current state. It is mostly used to subjectively evaluate alertness and fatigue, with lower scores indicating lesser degrees of drowsiness and greater alertness.

### 2.4. Statistical analysis

Sample size estimates were for paired-samples, two-tailed level of significance at 0.05/3 = 0.0167 and 90% power (28). Assuming a 20% drop-out rate, we aimed to recruit *n* = 19 in the subgroups to have power to detect statistically significant differences in sleep with red lights. The statistical analysis in this study was performed using SPSS software (version 26.0). Categorical variables were analyzed using chi-square tests. Measurement data that followed a normal distribution were expressed as mean ± standard deviation values. Comparisons between groups were conducted using *t*-tests for two-group comparisons and one-way ANOVA followed by the Student-Neuman-Keuls (SNK) multiple test for comparisons among multiple groups, assuming homogeneity of variances. In cases where the variances varied, the rank sum test was used for analysis. The original data was then converted into rank variables, and the SNK test was performed for pairwise comparisons. The measurement data that did not conform to a normal distribution were expressed as median and interquartile-range values, with Wilcoxon rank-sum tests used for comparisons between two groups, and the Kruskal-Wallis H test used for comparisons between multiple groups. Partial correlation analysis was used to analyze the correlations between different variables. Mediation effect tests were performed using bootstrapping. All tests were two-sided, and *p* < 0.05 was considered significant.

## 3. Results

### 3.1. General data

The HS group comprised 24 males and 33 females with a median age of 29 years, Body Mass Index (BMI) of 21.40 ± 2.27 kg/m^2^, and median education duration of 16 years. The ID group comprised 22 males and 35 females with a median age of 36 years, BMI of 21.88 ± 2.62 kg/m^2^, and median education duration of 15 years. There were no significant differences in gender, age, BMI, or education durations between the HS and ID groups (Table 1) or between the subgroups (Table 1; *p* > 0.05).

**Table 1:** General data analysis of HS, ID group, and subgroups.

Variables	HS group	ID group		*χ^2^/t/Z/F*	*p* value
Gender (Male/Female)	24/33	22/35		0.146	0.703
Age	29 (24, 47)	36 (28, 50)		−1.862	0.063
BMI (kg/m^2^)	21.40 ± 2.27	21.88 ± 2.62		−1.029	0.306
Education (Year)	16 (9, 19)	15 (9, 16)		−1.306	0.192
	ID-RL group	ID-WL group	ID-BC group		
Gender (Male/Female)	6/13	10/9	6/13	2.369	0.306
Age	36 (29, 53)	30 (27, 48)	46 (28, 50)	2.423	0.298
BMI (kg/m^2^)	21.14 ± 2.72	22.01 ± 2.74	22.48 ± 2.34	1.299	0.281
Education (Year)	15 (9, 20)	15 (11, 16)	9 (9, 20)	1.434	0.488
	HS-RL group	HS-WL group	HS-BC group		
Gender (Male/Female)	7/12	8/11	9/10	0.423	0.806
Age	29 (28, 50)	30 (27, 47)	29 (24, 36)	4.294	0.117
BMI (kg/m^2^)	21.72 ± 2.07	21.30 ± 2.01	21.19 ± 2.75	0.281	0.756
Education (Year)	17 (9, 20)	16 (8, 18)	16 (15, 18)	1.834	0.400

### 3.2. Data analysis baseline

**Figure 2:**
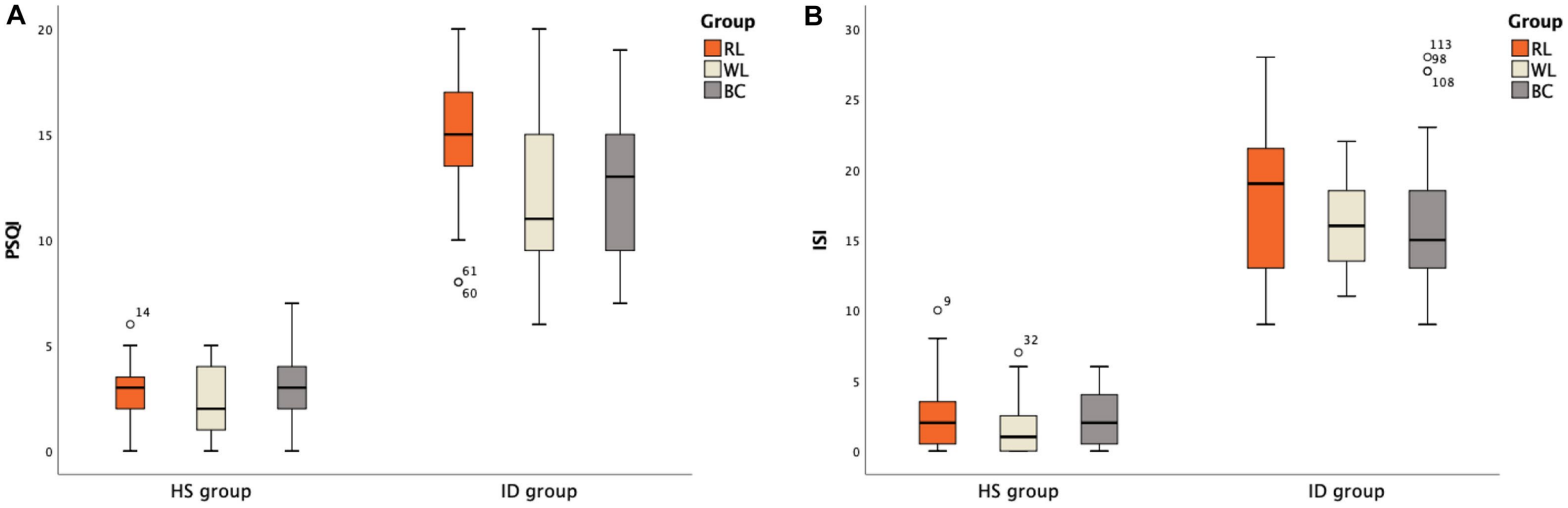
PSQI and ISI scale scores between groups: **(A)** PSQI scores between groups; **(B)** ISI scores between groups.

The Wilcoxon rank-sum test indicated that the PSQI score differed significantly between the ID (average rank = 85.94) and HS (average rank = 29.06) groups (Z = −9.209, *p* < 0.001; Figure 2A). The Kruskal-Wallis *H* test indicated that there were no significant differences in PSQI scores between the HS (χ^2^ = 2.400, *p* = 0.301) and ID (χ^2^ = 4.969, *p* = 0.083) subgroups (Table 2). The ISI score differed significantly between the ID (average rank = 85.96) and HS (average rank = 29.04) groups (Z = −9.218, *p* < 0.001; Figure 2B). The Kruskal-Wallis *H* test indicated that the ISI scores did not differ significantly between the HS (χ^2^ = 1.226, *p* = 0.542) and ID (χ^2^ = 1.607, *p* = 0.448) subgroups (Table 2).

**Table 2:** Comparison of ISI, PSQI, SAS, and SDS scores between the HS and ID group.

Subgroups	HS group		ID group	
	ISI	PSQI	ISI	PSQI
RL group	2.00 (0.00, 4.00)	3.00 (2.00, 4.00)	19.00 (12.00, 22.00)	14.58 ± 3.44
WL group	1.00 (0.00, 3.00)	2.00 (1.00, 4.00)	16.00 (13.00, 19.00)	12.05 ± 3.97
BC group	2.00 (0.00, 4.00)	3.00 (2.00, 4.00)	15.00 (13.00, 19.00)	12.42 ± 3.53
χ^2^*/F*	1.226	2.400	1.607	2.653
*p value*	0.542	0.301	0.448	0.080
Scales	HS group	ID group	*Z*	*p* value
SAS	30.00 (28.00, 35.00)	41.00 (34.50, 48.00)	−6.031	<0.001^*^
SDS	33.00 (29.00, 40.00)	44.00 (36.00, 51.00)	−5.180	<0.001^*^

^*^indicates statistically significant difference (*p* < 0.05).

There were significant differences in SAS and SDS scores between the HS and ID groups (Z = −6.031, *p* < 0.001 and Z = −5.180, *p* < 0.001, respectively; Table 2). There were no significant differences among the SAS and SDS scores of the subgroups (*p* > 0.05).

### 3.3. Data analysis of lighting intervention

#### 3.3.1. Emotion and alertness assessment

There were no significant differences in SAS and SDS scores among the HS subgroups (χ^2^ = 5.163, *p* = 0.076 and *F* = 2.853, *p* = 0.066, respectively). However, significant differences were observed in SAS scores among the ID subgroups (*F* = 5.426, *p* = 0.007). After conducting SNK test multiple comparisons, it was found that the mean SAS score of the ID-RL group (46.63 ± 7.06) was higher than that of the ID-WL group (38.74 ± 7.47) and ID-BC group (41.42 ± 7.98), and this difference was statistically significant. However, there was no statistical difference between the ID-WL group and ID-BC group (*p* = 0.276). No significant differences were found in the mean SDS scores among the ID subgroups (*F* = 3.067, *p* = 0.055; Table 3). One-way ANOVAs revealed significant differences in SAS scores between the post-intervention and baseline measurements in the ID-RL (average rank = 26.63) and HS-RL (average rank = 12.37) groups (Z = −4.005, *p* < 0.001; Table 3).

**Table 3:** Comparison of SAS, SDS, and KSS scores between the HS and ID subgroups.

	HS group			ID group		
	SAS	SDS	KSS	SAS	SDS	KSS
RL group	33.00 (27.00, 37.00)	35.74 ± 6.85	2.00 (2.00, 4.00)	46.63 ± 7.06	48.42 ± 9.06	4.00 (3.00, 5.00)
WL group	28.00 (26.00, 33.00)	31.79 ± 5.00	5.00 (3.00, 6.00)	38.74 ± 7.47	41.95 ± 8.24	6.00 (4.00, 7.00)
BC group	27.00 (30.00, 35.00)	36.00 ± 6.26	7.00 (5.00, 8.00)	41.42 ± 7.98	42.74 ± 9.05	8.00 (6.00, 9.00)
χ^2^/*F*	5.163	2.853	24.780	5.426	3.067	24.766
*p* value	0.076	0.066	<0.001^*^	0.007^*^	0.055	<0.001^*^

^*^indicates statistically significant difference (*p* < 0.05).

The HS group (average rank = 2847.00) had significantly lower KSS scores compared to the ID group (average rank = 3708.00; Z = −2.459, *p* = 0.014). Significant differences were also observed in the KSS scores among the HS (χ^2^ = 24.780, *p* < 0.001) and ID (χ^2^ = 24.766, *p* < 0.001) subgroups (Table 3).

The positive emotion score of the PANAS did not differ significantly between the HS and ID groups (*t* = 0.164, *p* = 0.870), while the negative emotion score was higher in the ID group than in the HS group (Z = −2.135, *p* = 0.033). There were significant differences in positive (*F* = 10.816, *p* < 0.001) and negative (*F* = 27.087, *p* < 0.001) emotions among the three HS subgroups. After conducting SNK tests for multiple comparisons, it was found that the positive emotion scores of the HS-RL group (30.32 ± 2.95) and HS-WL group (28.21 ± 3.43) were higher than those of the HS-BC group (25.21 ± 3.78; *p* < 0.05). However, there was no significant difference between the HS-RL group and HS-WL group (*p* = 0.062). Similarly, statistical differences were observed in negative emotions among the three HS groups (*F* = 27.087, *p* = 0.000). After conducting SNK tests for multiple comparisons, it was found that the negative emotion scores significantly differed among the HS-RL (25.42 ± 3.04, highest), HS-WL (19.89 ± 2.13), and HS-BC (19.63 ± 2.95) groups. However, there was no significant difference between the HS-WL group and the HS-BC group (*p* = 0.768).

Significant differences were also observed in positive (*F* = 5.534, *p* = 0.007) and negative (*F* = 55.597, *p* < 0.001) emotions among the ID subgroups. After conducting SNK tests for multiple comparisons, it was found that the positive emotion scores significantly differed between the ID-RL (29.89 ± 3.46) and ID-BC (25.84 ± 4.14) groups (*p* < 0.05). However, there was no significant difference between the ID-RL group and the ID-WL group (27.63 ± 3.66, *p* = 0.069), or between the ID-WL group and ID-BC group (*p* = 0.149). Regarding negative emotions, the scores in the ID-RL group (30.26 ± 3.66) were higher than those in the ID-WL group (20.53 ± 2.74) and ID-BC group (21.05 ± 3.14). However, the difference between the ID-WL group and ID-BC group was not statistically significant (*p* = 0.614).

**Figure 3:**
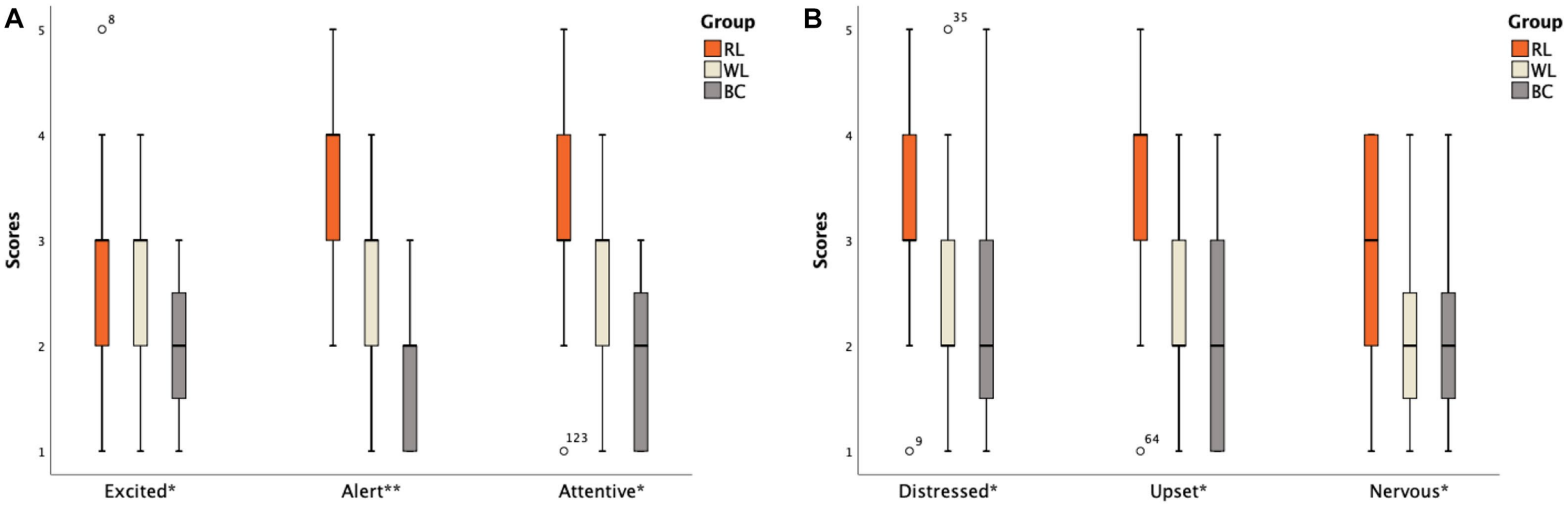
Scores of different items on the PANAS scale between the HS subgroups: **(A)** Positive emotion item; **(B)** Negative emotion item (The abscissa ^*^ indicates that there is a statistically significant difference between one of subgroups and the other two groups; ^**^ indicates that there is a statistical difference between any two groups among subgroups).

**Figure 4:**
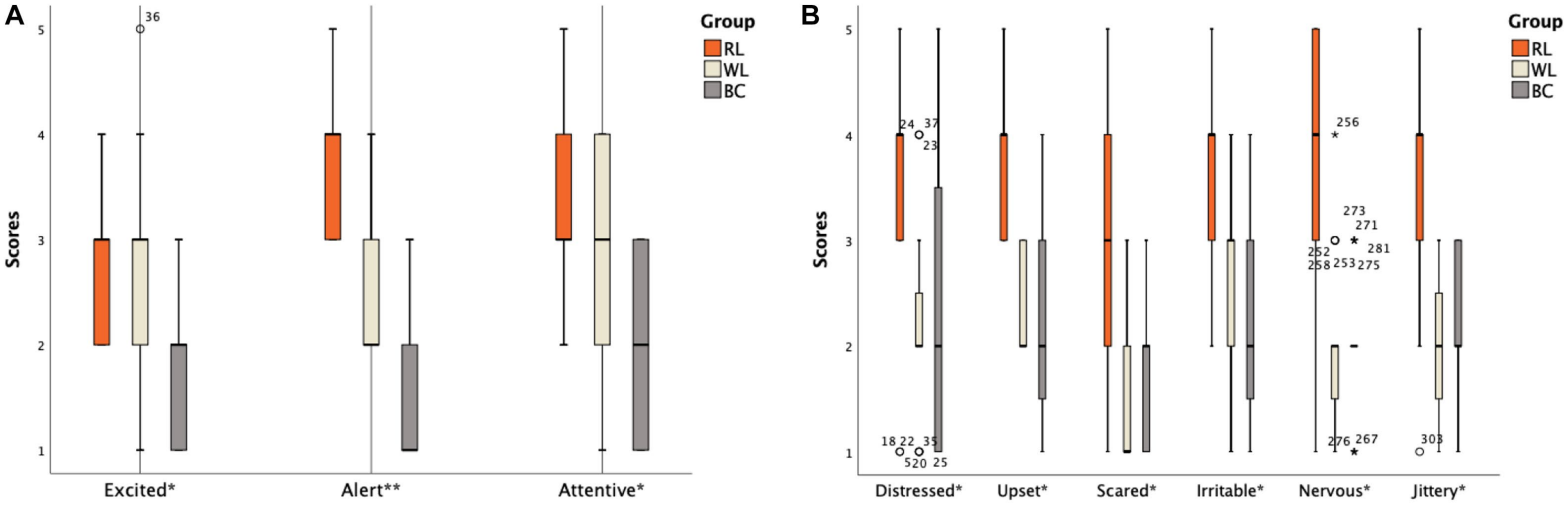
Scores of different items on the PANAS scale between the ID subgroups: **(A)** Positive emotion items; **(B)** Negative emotion items (The abscissa ^*^ indicates that there is a statistically significant difference between one of subgroups and the other two groups; ^**^ indicates that there is a statistical difference between any two groups among subgroups).

The differences in scores for each item in the PANAS scale were specifically analyzed. In the HS group, two items in the HS-BC group (Excited and Attentive) scored lower than the other two groups (*p* < 0.05). The order of scores for the Alert item was HS-RL group > HS-WL group > HS-BC group (Figure 3A). For negative emotions, three items in the HS-RL group (Upset, Distressed, and Nervous) scored higher than the other two groups (*p* < 0.05). None of the other items showed statistical significance (*p* > 0.05; Figure 3B). In the ID group, two items in the ID-BC group (Excited and Attentive) scored lower than the other two groups (*p* < 0.05) for positive emotion items. The order of scores for high alertness items was ID-RL group > ID-WL group > ID-BC group (Figure 4A). For negative emotion items, the items Upset, Scared, Distressed, Irritable, Nervous, and Jittery in the ID-RL group scored higher than the other two groups (*p* < 0.05; Figure 4B). There was no statistically significant difference for the other items (*p* > 0.05).

#### 3.3.2. PSG data

Compared to the HS-BC group, the HS-WL group demonstrated longer SOL (χ*^2^* = 9.780, *p* = 0.008), WASO (χ^2^ = 16.580, *p* < 0.001), and higher NWAK (*F* = 3.399, *p* = 0.041). The HS-BC group had significantly higher TST (*F* = 8.115, *p* = 0.001) and SE (χ^2^ = 23.108, *p* < 0.001) than both the HS-RL and HS-WL groups. The MAI was higher in the HS-RL and HS-WL groups than in the HS-BC group (*F* = 3.872, *p* = 0.027). Additionally, the NS-BC group had a lower N1% compared to the NS-WL and NS-RL groups (χ^2^ = 11.129, *p* = 0.004). There was no significant difference among HS subgroups in other parameters statistically (*p* > 0.05; Table 4).

**Table 4:** Differences of PSG parameters between HS and ID subgroups.

	HS-RL group	HS-WL group	HS-BC group	*F/χ^2^*	*p* value
SOL (min)	4.50 (2.00, 7.00)	9.50 (5.50, 15.50)	4.50 (2.50, 6.50)	9.780	0.008^*^
TST (min)	386.34 ± 37.40	395.74 ± 57.24	446.24 ± 51.12	8.115	0.001^*^
SE (%)	88.50 (85.50, 91.50)	84.80 (80.60, 87.80)	93.70 (92.00, 95.20)	23.108	<0.001^*^
WASO (min)	50.00 (27.00, 64.00)	61.5 (45.50, 73.00)	24.00 (17.00, 36.50)	16.580	<0.001^*^
NWAK (times)	27.26 ± 9.96	29.00 ± 11.02	21.58 ± 5.68	3.399	0.041^*^
NMA (times)	53.05 ± 17.68	54.00 ± 25.93	44.32 ± 13.85	1.381	0.260
MAI	8.23 ± 2.42	8.19 ± 3.89	5.98 ± 1.84	3.872	0.027^*^
N1%	11.30 (8.30, 13.90)	12.60 (8.60, 16.10)	6.60 (5.20, 8.60)	11.129	0.004^*^
N2%	45.40 ± 6.25	45.65 ± 7.11	45.22 ± 5.28	0.022	0.978
N3%	21.79 ± 7.59	21.31 ± 7.27	24.32 ± 6.78	0.954	0.392
R%	21.46 ± 5.94	20.63 ± 5.504	22.76 ± 4.51	0.766	0.470
Rem period number of cycles (number)	5.00 (3.00, 5.00)	4.00 (3.00, 5.00)	4.00 (4.00, 6.00)	1.038	0.595
Rem period SOL (min)	84.00 (69.50, 121.00)	95.00 (65.00, 152.00)	80.50 (64.00, 106.50)	1.158	0.560
Rem period NMA (times)	8.00 (5.00, 13.00)	12.00 (2.00, 16.00)	7.00 (4.00, 17.00)	0.112	0.946
Rem period MAI (times)	5.60 (3.90, 8.70)	5.90 (1.50, 10.30)	4.20 (2.80, 7.10)	0.667	0.717
	ID-RL group	ID-WL group	ID-BC group	*F/χ* ^*2*^	*p* value
SOL (min)	12.50 (10.50, 19.50)	25.50 (18.00, 42.50)	7.50 (3.00, 10.00)	24.437	<0.001^*^
TST (min)	401.03 ± 58.83	341.21 ± 71.49	395.32 ± 71.50	4.539	0.015^*^
SE (%)	79.35 ± 9.83	72.19 ± 12.17	88.49 ± 5.43	13.879	<0.001^*^
WASO (min)	89.18 ± 52.30	99.79 ± 51.54	43.68 ± 23.57	8.516	0.001^*^
NWAK (times)	32.95 ± 11.76	26.95 ± 10.89	26.47 ± 12.55	1.792	0.176
NMA (times)	53.21 ± 19.34	58.42 ± 24.58	43.68 ± 19.92	2.316	0.108
MAI	8.29 ± 3.67	10.56 ± 4.39	6.57 ± 2.70	5.709	0.006^*^
N1%	13.30 (7.50, 17.20)	11.60 (7.90, 17.70)	8.40 (6.10, 14.10)	2.906	0.234
N2%	51.21 ± 11.55	48.95 ± 9.68	47.88 ± 7.46	0.583	0.562
N3%	17.35 ± 7.48	21.89 ± 8.89	20.05 ± 6.85	1.639	0.204
R%	18.92 ± 5.98	16.42 ± 7.14	21.95 ± 5.15	3.861	0.027^*^
Rem period number of cycles (number)	5.00 (4.00, 7.00)	3.00 (3.00, 4.00)	4.00 (4.00, 5.00)	13.548	0.001^*^
Rem period SOL (min)	92.50 (71.50, 153.00)	99.00 (77.00, 153.50)	68.5 (61.50, 135.00)	2.291	0.318
Rem period NMA (times)	15.00 (8.00, 19.00)	8.00 (2.00, 11.00)	9.00 (3.00, 13.00)	7.157	0.028^*^
Rem period MAI (times)	8.14 ± 3.773	7.28 ± 4.174	5.91 ± 3.818	1.556	0.220

^*^indicates statistically significant difference (*p* < 0.05).

Insomnia disorder groups showed significant differences in SOL (χ^2^ = 24.196, *p* < 0.001), with the ID-WL group having the longest duration, followed by the ID-RL group, and ID-BC group. SE (*F* = 13.879, *p* < 0.001) showed the opposite pattern, with the ID-BC group having a higher sleep efficiency than the ID-RL group, which in turn was higher than the ID-WL group. Compared to the other two groups, the ID-BC group had a longer TST (*F* = 4.539, *p* = 0.015) and shorter WASO (*F* = 8.516, *p* = 0.001). Compared to the ID-WL group, the ID-BC group has a smaller MAI (*F* = 5.709, *p* = 0.006) and a higher R% (*F* = 3.861, *p* = 0.027). Additionally, number of cycles (χ^2^ = 13.548, *p* = 0.001) and NMA during the REM period (χ^2^ = 7.157, *p* = 0.028) were higher in the ID-RL group than that in the ID-WL and ID-BC groups (Table 4).

### 3.4. Correlation analysis of emotion, alertness, and PSG parameters

The correlations between scores of emotion scales, alertness scales, and sleeping parameters were considered partial based on the impact of demographic variables (age, gender, BMI, and education duration). The results indicated that SOL and NWAK were positively correlated with SAS, SDS, and negative emotion scores; SE was negatively correlated with SAS and SDS scores, REM period NMA and MAI were positively correlated with SAS and negative emotion scores, while KSS scores and sleeping parameters were not significantly correlated (Table 5).

**Table 5:** Correlation analysis of emotion, alertness and sleeping parameters.

	SAS	SDS	KSS	Positive emotions	Negative emotions
SOL (min)	0.440 ^*^	0.331^*^	0.113	0.062	0.351^*^
TST (min)	−0.004	0.030	0.148	−0.096	0.068
SE (%)	−0.244^*^	−0.200^*^	0.069	−0.080	−0.169
WASO (min)	0.180	0.164	−0.095	0.057	0.127
NWAK (times)	0.227^*^	0.241^*^	−0.094	0.098	0.221^*^
NMA (times)	0.104	0.112	−0.129	−0.106	0.084
MAI	0.107	0.107	−0.143	−0.055	0.066
N1%	0.057	0.076	−0.141	0.048	0.040
N2%	0.111	0.094	−0.022	0.073	0.071
N3%	−0.066	−0.083	0.058	−0.019	−0.116
R%	−0.108	−0.074	0.086	−0.128	0.024
Rem period number of cycles (number)	0.010	0.082	0.081	0.014	0.143
Rem period SOL (min)	0.123	0.139	−0.150	−0.039	0.028
Rem period NMA (times)	0.216^*^	0.143	0.044	−0.034	0.298^*^
Rem period MAI (times)	0.247^*^	0.113	−0.003	0.020	0.250^*^

^*^indicates statistically significant difference (*p* < 0.05).

### 3.5. Analysis of the mediating effect of emotion

We examined the potential mediating effect of negative emotion. The mediation effect analysis procedure (29) involved two dummy variables with red light as the reference (independent variable), and SOL (dependent variable), negative emotion (mediator variable), and demographic variables (control variable). The bootstrapping method was used to test the mediation effect (30). The results indicated that the mediation model was a significant predictor (*p* < 0.05; Table 6), which suggests that negative emotions play a partial mediating role (Table 6).

**Table 6:** The mediating effect test of negative emotions the ID group.

	*R*	*R^2^*		Adjusted *R^2^*	*F*	*p*
Model 1	0.845	0.715		0 0.680	20.867	<0.001
Model 2	0.851	0.724		0 0.684	18.313	<0.001
ID group	Effect	SE (boot)	T	*p*	95% CI	
					LLCI	ULCI
WL group vs. RL group	−37.626	9.697	−8.976	<0.001	−59.768	−21.879
BC group vs. RL group	−33.768	9.404	−8.027	<0.001	−54.146	−18.985

Model 1: variables to mediating variables; Model 2: variables to mediating variables to dependent variables.

## 4. Discussion

We report here that even being exposed to 75-lx red light for 1 h before bedtime has an impact on sleeping. The effects of red light are not limited to sleep, but also affect mood and alertness. Specifically, PSG parameters and scores on the KSS and PANAS were lower among participants with insomnia symptoms, indicating influences in sleeping, mood, and alertness.

We observed that subjective sleep quality was worse and the degree of dissatisfaction with sleep patterns and daytime functional impairment were greater in individuals with insomnia disorder than in healthy people (31, 32). At the same time, individuals with insomnia experienced anxiety and depression more often than the healthy population, which is consistent with previous research (33, 34). Insomnia, anxiety, and depression often coexist and interact with each other. There is evidence that sleep deprivation leads to reduced availability of dopamine D2/D3 receptors in the striatum and serotonin transporter gene expression dysregulation (35, 36). However, dopamine and serotonin play important roles in the mechanisms of depression and anxiety. Decreased dopamine content is closely related to anhedonia in depression (37). Short serotonin allele mutations lead to reduced serotonin uptake and increased susceptibility to anxiety and depression (38). Anxiety and negative mood also occur significantly more often after a red-light intervention in people with insomnia, and they tend to be emotionally unstable. Xie et al. (39) found that red-light exposure increased anxiety-like behaviors in mice. Previous research on color and emotion has also linked red light with negative events, with even children aged 5–10 years being prone to negative feelings under red-light backgrounds (40, 41), also people with strong anxiety traits commonly had greater anxiety levels when faced with different color stimuli (42). El-Sheikh et al. (43) studied the relationship between emotion regulation and sleep problems and found that decreased vagal inhibition levels were indicators of worse emotion regulation. Chronic insomnia can affect cognition, and dysfunctional cognition internalizes negative emotions. People with poor sleep quality may have worse emotional stability (44) and are more likely than healthy individuals to have fluctuating anxiety levels and other negative emotions after receiving a red-light intervention.

We found that individuals with insomnia symptoms had higher levels of hypersomnia and decreased alertness, since insufficient sleep reduces alertness. Even though the intervention period was short, with only one night of light treatment, the data are insightful. Yang et al. (45) found that participants experienced a significant decrease in alertness after a night of sleep deprivation. Another study found that long-term shifts resulted in a decrease in average sleep time, which affected alertness on the following day (46). Chronic sleep deprivation (sleeping for less than 5.6 h per day) also impairs attention and reduces self-assessment of alertness (47), but people with relatively stable sleep durations maintain more consistent levels of work performance and alertness during the night (48). A red-light intervention can increase subjective alertness and improve drowsiness for participants with insomnia. Figueiro et al. (16) found that red light can improve the subjective and objective alertness of night shift workers without affecting melatonin secretion and sleep. Askaripoor et al. (13) found that red light can improve physiological alertness more than white light can. In summary, red light has been found to have specific advantages in alertness, improving it without affecting melatonin secretion; it may also improve the performance of night workers.

The present study is the first that we are aware of that employed red light as a factor to investigate objective sleeping and the relationship between sleeping parameters, emotion, and vigilance. Red light even affected sleep at 75 lx, which was consistent with previous findings (28). Tähkämö et al. (49) also suggested that even the longest red-light wavelength (631 nm) may induce resetting of circadian rhythms. Red light has specific advantages in sleep initiation when compared with white light. This may due to red light resetting the melatonin rhythm via visual photoreceptors (50), but there was no evidence indicating that red light increases melatonin secretion, which may be related to the finding that red light improves sleep initiation rather than sleep maintenance.

We also found changes in the REM period, such as increased number of cycles and NMA during REM period for people with insomnia after a red-light intervention, which was positively correlated with anxiety and negative emotion. A previous study found that experiencing negative emotion events before bedtime can fragment sleep, with especially the percentage of REM sleep decreasing and the number of awakenings during REM sleep increasing (51). Other previous studies found that rats subjected to REM sleep deprivation exhibited depressive symptoms, decreased pleasure-seeking behaviors, and increased alcohol preference (52, 53). The activity of some brain structures (e.g., the amygdala, cingulate gyrus, and dorsolateral prefrontal cortex) increase during REM sleep, and these areas also play crucial roles in emotion regulation and memory processing (53, 54). Changes in REM sleep may therefore be induced by red light before bedtime, which causes negative emotions in people with insomnia. We found that higher levels of anxiety, depression, and negative emotion before bedtime were associated with a longer SOL, lower SE, and more WASO during the night, and larger NMA during REM sleep; these observations were consistent with previous studies.

Negative mood can lead to decreased sleep quality, and poor sleep quality can increase the probability of negative mood (55–57). Orchard et al. (58) found that depression and anxiety disorders were accompanied by insufficient sleep and poor sleep quality in adolescents, and sleep parameters could predict the degree of depression and anxiety and whether the subject will subsequently suffer from anxiety or depressive disorders. In contrast, unlike previous predictions, the increase of subjective alertness caused by red light was not sufficient to affect sleep, and so the correct conclusion was that subjective alertness is time-sensitive, which needs further evidence from follow-up studies.

A black environment before bedtime was helpful in improving sleep quality (short SOL, increased TST and SE, and better sleep continuity) in both individuals with insomnia and healthy participants, especially for the R%. A previous study also found decreases in the REM period and the proportion in individuals with insomnia symptoms (59), and REM sleep is closely related to cognition, emotion, and many physical diseases. Changes in REM sleep can cause a decline in cognitive function and manifest as emotional regulation disorders (60, 61). Our study therefore suggests that people with ID should be exposed to appropriate light stimulation before bedtime, because reducing the amount of light can help restore the proportion of REM sleep.

In summary, we have explored the effects of red light on objective sleep structure, mood, and alertness in people with insomnia disorders. Red light can directly influence sleep, such as the fragmented changes in REM period, and negative emotion plays an important role in this process. Increased subjective alertness, anxiety, and negative mood may be responsible for changes in REM sleep under red light. These findings provide solid evidence for the selection of a control light level in biological experiments and reasonable lighting before bedtime, we will continue to test the biological role of red light in the follow-up trials.

## Data availability statement

The original contributions presented in the study are included in the article/supplementary material, further inquiries can be directed to the corresponding author.

## Ethics statement

The studies involving humans were approved by Ethics Committees in the First Affiliated Hospital of Jinan University. The studies were conducted in accordance with the local legislation and institutional requirements. The participants provided their written informed consent to participate in this study.

## Author contributions

RP and GZ were involved in the design and research methodologies and drafted the manuscript. FD and WL were involved in data curation and investigation. JP revised the manuscript. All authors contributed to the article and approved the submitted version.

## Funding

This work was also supported by a grant from National Key Research and Development Program of China (grant number: 2022YFC2503902).

## Conflict of interest

The authors declare that the research was conducted in the absence of any commercial or financial relationships that could be construed as a potential conflict of interest.

## Publisher’s note

All claims expressed in this article are solely those of the authors and do not necessarily represent those of their affiliated organizations, or those of the publisher, the editors and the reviewers. Any product that may be evaluated in this article, or claim that may be made by its manufacturer, is not guaranteed or endorsed by the publisher.

## Figures and Tables

**Table:** 

ID	Insomnia disorder
HS	Healthy subject
RL	Red light
WL	Write light
BC	Black control
ipRGCs	Intrinsically photoreceptor retinal ganglion cells
REM	Rapid eye movements
NREM	Non-rapid eye movements
PSG	Polysomnography
EEG	Electroencephalogram
EOG	Electrooculogram
EMG	Electromyogram
ISI	Insomnia severity index
PSQI	Pittsburgh sleep quality index
SDS	Self-rating depression scale
SAS	Self-rating anxiety scale
PANAS	Positive and negative affect scale
KSS	Karolinska sleepiness scale
AASM	American academy of sleep medicine
SOL	Sleep onset latency
TST	Total sleep time
SE	Sleep efficiency
NWAK	Number of awakenings
WASO	Wakefulness after sleep onset
NMA	Number of microarousal
MAI	Microarousal index
BMI	Body mass index
HS-RL	Healthy subject red light
HS-WL	Healthy subject write light
HS-BC	Healthy subject black control
ID-RL	Insomnia disorder red light
ID-WL	Insomnia disorder write light
ID-BC	Insomnia disorder black control
